# Application of Data Mining to a Large Hearing-Aid Manufacturer’s Dataset to Identify Possible Benefits for Clinicians, Manufacturers, and Users

**DOI:** 10.1177/2331216518773632

**Published:** 2018-05-31

**Authors:** Joseph Mellor, Michael A. Stone, John Keane

**Affiliations:** 1 School of Computer Science, University of Manchester, Manchester, UK; 2 Manchester Centre for Audiology and Deafness, University of Manchester, Manchester, UK; 3 Manchester Academic Health Sciences Centre, University of Manchester, Manchester, UK; 4 Manchester Institute of Biotechnology, University of Manchester, Manchester, UK

**Keywords:** audiogram, auditory ecology, big data, candidature, hearing aids

## Abstract

Modern hearing instruments contain logging technology to record data, such as the acoustic environments in which the device is being used and how the signal processing is consequently operating. Combined with patient data, such as the audiogram, this information gives a more comprehensive picture of the user and their relationship with the aid. Here, a relatively large, anonymized dataset (>300,000 devices, >150,000 wearers) from a hearing-aid manufacturer was data mined for connections between subsets of the logged varieties of data. Apart from replicating links that have previously been reported in labor-intensive studies, a link between device style (in-the-ear/behind-the-ear) and the sound levels of encountered environments was demonstrated, suggesting that some device types are more successful from a lifestyle perspective. Furthermore, the data also suggested links between the audiogram and the sound environments in which the aid was operated. Modeling the expected link between the environment and the microphone directionality settings revealed patterns of either abnormal fitting or where the aid was not operating correctly—factors that may indicate a failed fitting. Given the necessarily redacted nature of the dataset, the reported findings represent a proof-of-concept of the use of relatively large-scale data mining to guide and assess hearing-aid fitting procedures for possible benefits to the clinician, manufacturer, and patient.

## Introduction

There are many factors that contribute to a successful hearing-aid fitting, only some of which are related to the hearing loss or hearing aid. It has long been recognized that the audiogram is insufficient to describe the residual hearing capabilities (Hirsh, Rosenblith, & Ward, 1950) and hence outcomes with aiding. In a review of nearly 30 years of literature exploring the journey from first seeking an aid to a successful outcome, Vestergaard-Knudsen, Öberg, Nielsen, Naylor, and Kramer (2010) could only identify a single factor, the user’s self-perceived degree of hearing loss, that positively influenced the whole patient journey from first seeking to long-term use. They reported a variety of other factors that were responsible for positively influencing different parts of the journey (e.g., personality, amount of social interaction, and dexterity). The multiplicity of patient factors to be considered in a hearing-aid fitting, combined with the complexity of interactions possible within algorithms used in modern hearing aids, means that it is unlikely that relationships between patient factors and predicted usage of the aid will be correctly identified by the human observer, challenging the ability to set up the aid optimally from the time of first fitting.

In a landmark study attempting to establish relationships between factors additional to hearing loss affecting the benefits of aiding, Gatehouse, Naylor, and Elberling (2006a, 2006b) demonstrated links between lifestyle demands, cognitive abilities, and the acoustic environment in which the aid wearer expects to operate, that were predictive of candidature and outcome for a patient. For example, greater benefit from slow-acting dynamic range compression was associated with lower performance on a cognitive test. The study of Gatehouse et al. (2006a, 2006b) was rather laborious, involving many testing sessions, and was confined to a relatively small population of established hearing-aid users. The ability of a modern hearing aid to record, or log, its dynamic operation permits the automatic generation of large datasets from a wider sample of the population, and at a lower cost, than was possible with this sort of study.

Examples of the sort of data that are routinely logged are the general properties of the acoustic environment in which the aid is being used, the decisions as to how the aid classifies the acoustic features of the environment, and how the aid is processing that sound for the user.

An alternative approach to the focused study of Gatehouse et al. (2006a, 2006b) would be to use data mining techniques on a relatively large dataset, which could also include not just experienced but also many first-time, hearing-aid users. The purpose would be to identify important factors contributing to a successful fitting and their relationships for an individual patient and to capture changes in fittings as experience with the device increased. This alternative approach offers a more representative sample of the patient population.

In recent years, there has been an increase in the application of data mining to audiological data (Anwar & Oakes, 2011; Laplante-Lévesque, Nielsen, Jensen, & Naylor, 2014; Lee, Hwang, Hou, & Liu, 2010; Panchev, Anwar, & Oakes, 2013; Singh & Launer, 2016; Timmer, Hickson, & Launer, 2017). Most studies use small datasets (*N* = 1000), although two studies accommodated tens of thousands of users (Panchev et al., 2013; Singh & Launer, 2016). The earlier of these two studies is noteworthy in that it combined several different types of patient information with dispensing practice, increasing the dimensionality of the dataset. However, only a few studies have explored the data-logging facility of modern hearing-aid devices. These tend to focus on a single dimension in the logged data such as hours of use (Bentler & Nelson, 1997; Laplante-Lévesque et al., 2014; Marnane & Ching, 2015; Muñoz, Preston, & Hicken, 2014; Timmer et al., 2017) or volume control setting (Mueller, Hornsby, & Weber, 2008): There is a rich number of dimensions stored via data-logging that have been left relatively underexplored. One particular dimension in which we are ultimately interested is the change in usage patterns over time, which could be an indicator of successful uptake, or otherwise.

“Modality” is a standard term used to describe subsets of the data, by which is meant one or more interrelated dimensions of a particular kind. Often these interrelated dimensions are acquired via the same sensor (Lahat, Adali, & Jutten, 2015). For example, an audiogram is a collection of values of absolute threshold for a set of frequencies. The threshold at each frequency constitutes a single dimension, but any subset of the thresholds in the audiogram would be considered part of the audiogram “modality.” Similarly, the predicted output sound pressure level (SPL) for a single given frequency would constitute a dimension, but any subset of output levels would be considered part of the output SPL modality. As in Gatehouse et al. (2006a, 2006b), we are interested in the interplay *between* modalities, especially those outside of measures of hearing loss. In addition, we aim to identify how the interplay affects not just the hearing-aid wearer, as in the previously cited work, but also how it can provide guidance to the clinician and manufacturer.

This article reports the use of the Knowledge Discovery in Databases (KDD) process, briefly reviewed in relation to audiology in Mellor, Stone, and Keane (2018) or in more depth in general purpose works, such as Fayyad, Piatetsky-Shapiro, and Smyth (1996) and Han, Kamber, and Pei (2011), to analyze an anonymized, relatively large dataset provided by a hearing-aid manufacturer. To protect commercial confidentiality, and the anonymity of patient, clinician, and dispensing practice, the data fields supplied, as well as the level of detail within each field, were a subset of the actual data collected. In addition, some data collection that was omitted from transmission to us may have been for purely administrative purposes by the manufacturer and have no rational link to patient outcomes. Hence, the results reported here represent a demonstration of the capabilities of data mining methods to identify relationships between data collected across a multiple of dimensions, which may be of benefit to all stakeholders involved in the supply, fitting, and use of hearing aids. Given the redacted nature of the dataset, the results should be seen as a “proof-of-concept” of the capabilities of data mining, while at the same time highlighting certain pitfalls due to anomalies identified by analyses used on the dataset.

The article starts by describing the provenance of, and caveats associated with, the dataset. It then shows that the application of data mining techniques on a relatively large dataset provides analysis and insight that are compatible with previous findings reported from smaller but heavily curated datasets. After this, further analyses establish linkages between different data dimensions, such as the choice of device type or the audiogram and associated patterns of usage. Such linkages are reported in the context of how they have the potential to provide benefit to clinician, manufacturer, and patient.

## Details of the Dataset

The dataset comprised records for 316,758 individual devices attending a total of 979,289 fitting sessions, with 1,764,739 device “snapshots” (which reflect device configurations multiply recorded both within and across fitting session). The data were deidentified before being shared with the researchers and were therefore not subject to approval of an Ethics Panel (Institutional review board). Due to the anonymization process, we cannot identify exactly how many individual wearers these figures represent, since the data, as received, do not directly identify links between left and right devices on the same wearer. In addition, as shown later, some devices appear to have been lent to different users. A conservative estimate, after allowing for such “loan” devices, as well as assuming 100% binaural fittings, is in excess of 150,000 unique wearers. To the best of our knowledge, this is the largest hearing-aid related dataset that has been reported in the literature to date. The dataset represents the logging from the same generation of signal processing chip at the core of a hearing-aid function. The same function has been implemented in a variety of physical housings (e.g., behind-the-ear (BTE)/in-the-ear (ITE)). It should be recognized that the combination of this particular generation of hardware, and its associated fitting software, represents a snapshot of one company’s approach to hearing aids over a comparatively short time period. Therefore, the generalizability of the detail of any findings should be cautious in its application.

The data fields provided included:

- the audiogram,
- the proportion of time the device was in a given range of input level (12 categories, mapping from “ < 40 dB SPL,” then in 5-dB ranges, until the final category of “ > 90 dB SPL”), as measured by the device microphone,
- the proportion of time the device was in a different acoustic classification (e.g., quiet, noise, or speech),
- the degree of gain reduction in each acoustic classification,
- the output SPL delivered into a 2-cc coupler, independent of device style,
- the style of device (spanning several behind-the-ear and in-ear device constructions),
- the number of days since the last visit for a fitting,
- a unique serial number by which each device was identified, such that
- the chronology of fitting sessions could be established by a second unique serial number which incremented with time across the entire dataset.

These data fields can be subgrouped since some relate to (a) the wearer, (b) the acoustic environment in which they operate, and (c) the device behavior in response to the patient settings and acoustic environments.

**Figure 1.**
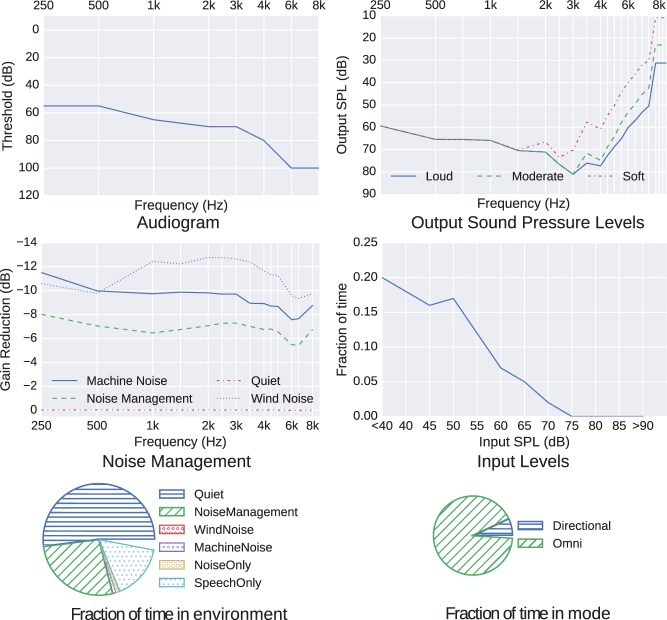
Example record from dataset.

Examples of these data are given in Figure 1.

Although labels for the data fields have been given earlier, some caution should be applied to their interpretation. For example:

- *Concerning the “audiogram” stored in the device*: The values recorded are those entered by the clinician into the fitting software in order to get the aid into a desired state, and hence might not reflect the exact audiogram of the aid wearer. An exploration of the extent of and handling of missing values is described later in connection with Table 1.
- *Concerning the objectivity implicitly attached to the data fields: A value in a data field should not be interpreted as being “optimal” for that patient*: No assumptions should be made that the data were indicative of an optimal fitting. One hypothesis is that, if data mining is to produce insight into the data, it should be able to identify not just beneficial behaviors but also those settings or behaviors that lead to negative or no benefit, such as nonoptimal settings. Part of the purpose of the analyses is to investigate the degree of appropriateness, as exhibited through other dimensions of the data.
- *Concerning the accuracy of classification of a sound scape*: To be implemented on such a low-power device, a compromise has to be made between complexity and accuracy of the acoustic classifier. The proportion of time spent in each class cannot therefore be 100% accurate, but we start with the presumption that the aid designer had made them “sufficiently accurate” to be useful.
- *Concerning the calibration of the devices such as the measures of Input SPL and Output SPL*: Given the precision inherent in digital processing, these values could be expected to remain in relative calibration and therefore form an ordinal, rather than a cardinal, scale. However, their absolute calibration relies on the hearing device remaining within manufacturer’s tolerances during the period of data collection. Any calibration drift will reduce their accuracy and add “noise” to the logged data, reducing the discovery of, and likely strength of, any relationships related to these dimensions. Manufacturers recommend regular servicing of their devices, such as by use of replaceable microphone covers, or disposable wax traps at the ports of receivers placed in the auditory canal, so as to limit the effect of these sources of drift. If these service intervals are not observed, then erroneous data could result.
- *Concerning the comparison of measures of “InputSPL” across devices*: Input SPL is referenced to being measured at the device microphone, with no correction for device style. Acoustic diffraction effects around the pinna mean that the frequency response differs between device types due to the difference in microphone position (Bentler & Pavolovic, 1989). When comparing the total power received at the microphone from a free-field source with a “white-noise” (i.e., flat) spectrum with a 250 to 6300 Hz bandwidth, the maximum difference between a behind-the-ear device and an in-ear or in-canal device was 2 dB. For more realistic environmental sounds, such as speech, the magnitude of the difference was 1.5 dB or less. Given the 5 dB size of the categories in the “InputSPL” modality, these differences are unlikely to effect major changes in histogram shape when comparing across device styles.

**Table 1.** The Distribution of Records by Pattern of Audiogram Missing Values in a Dataset, Ranked by Frequency of Occurrence Within the Dataset.

Frequency (kHz)	0.25	0.5	1	2	3	4	6	8
Occurrence rate (%)								
60.13								
11.40								*✗*
9.55					*✗*		*✗*	
7.24							*✗*	
4.86					*✗*			
2.20							*✗*	*✗*
1.44					*✗*			*✗*
1.06					*✗*		*✗*	*✗*
0.46	*✗*							
0.43	*✗*							*✗*
0.43	*✗*	*✗*	*✗*	*✗*	*✗*	*✗*	*✗*	*✗*
0.24	*✗*				*✗*		*✗*	*✗*
0.17	*✗*				*✗*		*✗*	
0.15	*✗*						*✗*	*✗*
0.09	*✗*						*✗*	
0.08	*✗*				*✗*			
0.07	*✗*				*✗*			*✗*

*Note.*A cross marks the missing values. The majority of fittings (60% of total data) record values for each frequency in the audiogram, while the next most common type of fitting (11%) does not record the value at 8 kHz. In a small number of records (0.43%), no values are recorded at all.

Use of such a large dataset overcomes some of the limitations of laboratory experiments which typically use small numbers of usually highly motivated users. However, the dataset was collected from a population that attended audiology clinics, which was still a subset of the total population. Any representative capture of the “real-world” population requires expensive epidemiological studies.

Other limitations of this particular dataset concerned the need for anonymization and commercial confidentiality. The dataset contained no demographic information as to the age or gender of the aid wearer or on which ear the device was worn. In addition, the measures of timing of device use were only given as proportions and not absolute units such as hours. To perform some of the reported analyses, we have therefore had to make assumptions in order to select (“preprocess”) the data to use those which are likely representative only if the device has been used for a sufficiently long period of time. Finally, for the sake of impartiality, our relationship with the manufacturer meant that, apart from discussions over definition of the data fields and some small details of clinical practice and fitting procedures, our analysis was largely “blind” and not directed by the manufacturer. We chose which analyses to perform and report.

## Data Preprocessing

As mentioned in Mellor et al. (2018), individual records of datasets can be noisy or anomalous in a multitude of ways. As an example, Table 1 shows the distribution of missing values in the audiograms recorded across our dataset; 35.6% of records have only one or two missing values. Interpolation could be used to impute some missing values, and the erroneous records reincluded in the dataset. Other potential sources of data unreliability were also identified, as detailed later, which were less amenable to interpolation. Hence, for the purpose of this proof-of-concept study, we chose an aggressive form of preselection in order to isolate what we estimated to be reliable parts of the dataset. As a first level of data cleaning, we therefore rejected dataset records:

- *if the number of days since the last visit was 7 or fewer*: For such a short usage duration, it was unlikely that the device had been used in a sample of acoustic scenarios representative of typical use.
- *if the sum of proportions of time in the modalities of “InputSPL” or acoustic classification did not approach unity*: The time proportions were specified to two significant figures. Therefore, in scenarios of low usage, the sum of the proportions would suffer large quantizing errors and not add up to unity. Any record where this sum was less than 0.9 was dropped from analysis.
- *if there were missing entries for any of the audiogram values*: The routine omission of measurements, such as at 3 and 6 kHz, may be a regular practice of either certain clinicians or certain clinics. Omission of these data may introduce a bias into analyses, but in the absence of any further identifiers in our dataset, we could not check for the existence of, or magnitude of, such a bias.
- *if other characteristics of a dataset member indicated questionable provenance*: Examples of these characteristics will be given later, according to the analysis performed.

It should be noted that while Rules (i) and (ii) were rigidly applied to provide a primary level of cleaning, some of these rules were not applicable in some analyses. For example, if an analysis did not require information about the audiogram, then Rule (iii) was not applied to the dataset records used in the analysis. Application of these secondary cleaning rules (e.g., iii and iv) meant that the size of the datasets varied between analyses. Subsidiary cleaning rules may have been necessary once anomalous behavior was detected, even after the secondary level of cleaning. We describe one such example later, the detection of loan devices. This iterative selection and preprocessing is an example of the recursive nature of the KDD process described in Mellor et al. (2018). We therefore detail below the dataset size used in each analysis.

## Example Applications of Data Mining

### Identifying Anomalous Data Series

In general, measures in most dimensions for a device are expected to be correlated over successive visits since a device is normally only used by one person, and, except for some audiological conditions, dramatic changes in data, such as the audiogram, are uncommon. For a number of devices, we identified that, over successive visits, the recorded audiogram changed by amounts greater than would be expected from variability arising from test or retest. One possible explanation was that the data came from a loan device, such as when a repair was being made to a wearer’s regular device. The identification of such loan devices would be an important step when trying to identify change in device or wearer behavior from a time series of device snapshots: Unless the device data were synonymous with that generated by the same wearer, such a data series would be meaningless.

We present here a conservative estimate of the extent of this practice of loaning of devices in this dataset. Some loan devices were obvious to spot due to associated large improvements in the audiogram over time. Since users are rarely likely to experience improved hearing over time, we conclude that the device was being worn by multiple people. We first removed all records for which there were missing values in the audiogram. Devices where thresholds improved by 15 dB or more at three or more frequencies, and where the time since the last visit was in excess of 7 days and less than 60 days, were excluded. Such a widespread general improvement in the audiogram is not generally expected. The 7-day threshold was chosen so that the device could have had a modest, representative, amount of use, and the 60-day upper limit was small enough so that short-term loan devices were more likely to be represented.

Such “hard” thresholds may remove legitimate instances but allows the change between audiograms to be ascertained in a standardized way.

Using this selection procedure, we list in Table 2 the percentage of devices meeting these criteria for each device style. Since the completely-in-the-canal (CIC), HalfShell, ITE, and ITC devices require a mold of the ear, they are highly unlikely to be loan devices. Using Fisher’s Exact Test, we find that the percentage of identified CIC, HalfShell, and ITE devices are not significantly different to one another at the 5% level (if *p* ≤ .05).

**Table 2.** Percentage of Devices Identified by Large Changes in the Audiogram Between Successive Visits, as a Function of Device Style.

	% identified	Number of occurrences
CIC	1.4	87
HalfShell	0.5	4
ITE	1.2	21
ITC	3.5	99
BTEa	10.7	2375
BTEb	9.3	405

*Note.* CIC = completely-in-the-canal; ITC = in-the-canal; BTE = behind-the-ear; ITE = in-the-ear. Other abbreviations as before. There were two variants of BTE devices, BTEa and BTEb.

That is, for each device in this group of three, there is no other device which is significantly different in the rate at which the anomalous audiogram behavior occurs, whereas there is a significant difference between rates for these devices and for any of ITC, BTEa, and BTEb.

The identification of these devices, unloanable due to their custom fitting, at an average level of 1% to 2%, hints at some other practice in the setting of the audiogram, such as an alternative way of fitting the device. We verified with the manufacturer that such practice was not intended in the fitting software, indicative of off-protocol use affecting around 1% to 2% of in-ear devices. If this (abnormal) fitting practice occurs at a similar level in the BTE style devices, we would expect and estimate of true “loan” devices to be around (10% to 2%) = 8%. The exact number, and its interpretation, depends on the strictness of the thresholds used in the selection criteria described earlier.

### Replication of Lee et al. (2010): Clustering to Find Generic Audiogram Shapes

The dataset analyzed here is typical of many such sets in that its generation was not the result of a focused hypothesis or research question; hence, its analysis is known as a secondary data analysis (Glass, 1976). If data mining is to supplement or replace (at least some) existing experimental methods, there is a need to show the ability to replicate previously accepted results. Unresolvable differences may teach caution in the use and interpretation of data mining results from such a broad-spectrum approach.

The study of Lee et al. (2010) used a hand-curated data set to identify a small number of generic shapes of the audiogram. Another part of the curation was that patients aged under 30 were excluded from their study. Further details of the curation are shown in the left-hand column of Table 3. For comparison, the same details from our dataset are shown in the right-hand column of this table. As can be seen, even for the conditions in which values are given, the two populations are very different. This is not unexpected, since Lee et al. (2010) focused on a very specific subset of the population, chosen for their sensorineural hearing impairment, while our dataset focuses on hearing-aid wearers from a wider population with hearing impairment, and hence likely mixed losses. Analysis of the two datasets might then be expected to produce very different results. In this section, we show how this can be the case, but despite the extent of the unknowns detailed in Table 3, and with the application of *a priori* knowledge, we can produce evidence for similarities between their dataset and a selection of our dataset. Despite the difference in collection methods, the same statistical “signal” is therefore present in both sets, but initially is lost in the variability of our large dataset.

**Table 3.** Comparison of the Datasets of Lee et al. (2010) to That Used in This Study.

	Lee et al.	This study
Data size, *N*	1,633	162,540
Male/female	719/914	NK
Type of losses	sensorineural, presbyacusic (screened)	mixed (unscreened)
Mean male age (years)	59.8 ± 12.4	NK
Mean female age (years)	58.0 ± 11.2	NK
Mean male hearing levels (0.25-8 kHz) (dB HL)	37 ± 23	NK
Mean female hearing levels (0.25-8 kHz) (dB HL)	26 ± 19	NK
Grand mean hearing levels (0.25-8 kHz) (dB HL)	31 ± 22	54 ± 14

*Note.* NK = not known.

The analysis reported by Lee et al. (2010) used the method of K-means (Han et al., 2011, pp. 451–454), and in their Figure 3, they settled on 11 as an optimum number to specify generic shapes. They transformed their audiogram shapes by offsetting all audiograms to a value of 0 dB HL at 250 Hz. Effectively, they were looking at clustering the changes in, rather than the absolute values of, the audiogram shape. These shapes ranged from near-flat (their “1” and “2”), gently sloping (“6” and “8”), and one (“11”) with a very steep loss between 250 and 1000 Hz, but near-flat and profound in level above 1 kHz. In addition, included were two (“4” and “9”), that were near-flat up to 2 kHz, but with a characteristic hump around 4 to 8 kHz, which, given their preselection of sensorineural losses, would generally be indicative of, at least, a component of noise-induced hearing loss (NIHL). In a large cohort of USA adults, the overall prevalence (bilateral and unilateral) of high-frequency hearing loss was higher in noise-exposed than nonnoise exposed individuals (means 43% compared with 27%), but barely different due to leisure-time noise (35% compared with 30%, respectively; Agrawal, Platz, & Niparko, 2008). Therefore, the characteristic NIHL dip in the high-frequency portion of the audiogram can reasonably be expected to be seen in some clusters, provided that we are searching for a sufficient number of clusters. For the purpose of an example, we use the two presumed NIHL shapes (“4” and “9” in Figure 3 of Lee et al., 2010) as an indicator of the statistical signal for which we are searching to replicate in our dataset.

**Figure 2.**
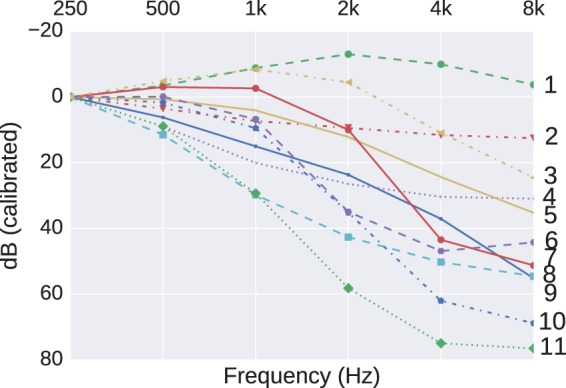
The 11 cluster centers found using K-means clustering.

Using our 100-fold larger dataset of 162,540 audiograms, a typical set of 11 cluster centers found by the K-means method is shown in Figure 2. The descriptor “typical” is used because the clusters are generated via a stochastic process to minimize a K-means objective. Changes in starting conditions do result in changes to the clusters found, but, once averaged across multiple starts, the cluster pattern is representative of these starts. The transformation used by Lee et al. (2010), to offset all audiograms to a value of 0 dB HL at 250 Hz, is reflected in our, and their, use of the ordinate label in Figure 2 of “dB (calibrated),” rather than dB (HL). Therefore, Cluster 1 in Figure 2, showing five audiogram values less than 0 dB, does not correspond to “Normal Hearing” since any such audiograms had been removed in the prior data cleaning (Mellor et al., 2018). The characteristic 4 kHz NIHL notch does not appear in our initial analysis (Figure 2). However, this may be explained by the previously identified differences between the two datasets, for example, the bias toward otologically abnormal patients of indeterminate age in our dataset.

**Figure 3.**
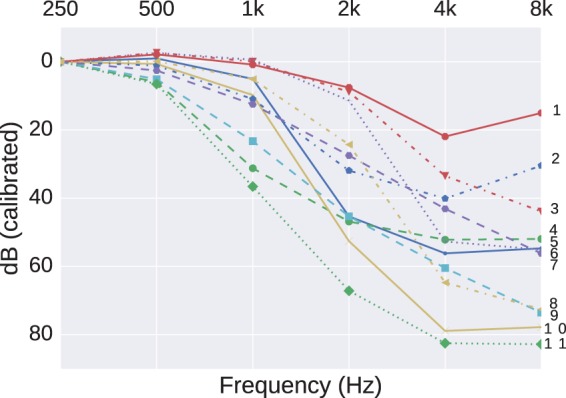
The 11 cluster centers found using K-means clustering on audiograms with a PTA (250,500) less than 20 dB HL.

To redress this bias, we screened for audiograms that were more likely to have arisen from an otologically normal and younger population (thereby reducing the possible confound of presbyacusis as well as possible [large] conductive losses), to select preferentially for a subpopulation of our dataset where NIHL may have had a greater influence. Although clinically “Normal Hearing” is commonly defined for thresholds not exceeding 25 dB HL, we used a more conservative figure of not exceeding 20 dB HL in order to only include patients who were otologically normal for an age range approximating the lower half of those patients seen by Lee et al. (2010). The ISO 7029 standard (International Organization for Standardization, 2000) tabulates equations for the mean and standard deviation of hearing thresholds to be expected as a function of frequency and age in an otherwise otologically normal population. Using these equations, a low-frequency pure tone average (PTA) (averaged over 250 and 500 Hz) not exceeding 20 dB HL can be expected in at least 80% of the otologically normal population aged 55 or under. Although this introduces a bias in mean age compared with Lee et al. (2010), Robinson (1988) reported that the difference in mean thresholds between screened and unscreened populations can be described by an accelerated ageing of around 10 to 15 years in the unscreened population. Any residual bias introduced by this new stage of cleaning is also offset by a noncomplete elimination of conductive or mixed losses.

The K-means method was applied to this smaller, filtered, dataset now comprising 25,938 audiograms. The grand mean hearing levels (0.25-8 kHz; dB HL) for this filtered dataset is 38 ± 9 which is more in line with what was reported by Lee et al. (2010). The revised set of clusters is shown in Figure 3. Dips around 4 kHz, with better thresholds at lower and higher frequencies, can now be seen in Clusters “1” and “2” and to a much lesser extent in Cluster “6,” indicating that these shapes did exist in subgroups of our dataset but were rendered into invisibility by other biases or confounds which were easy to account for by reference to alternative data sources. However, there still remain differences between this revised clustering and that of Lee et al. (2010): Their steeply sloping cluster “11” is not replicated in Figure 3. Possible explanations for this may be either that the hearing-aid designs in our dataset are not intended to fit such extreme losses or such losses may be more representative of candidates for cochlear implants. For either possibility, we would not expect to see a hearing-aid fitted, and hence no record in our dataset.

Given the large amount of unknowns about the demographics of our dataset, it is of interest that *a priori* knowledge, in this case (International Organization for Standardization, 2000), can be used to reduce the variance introduced by the unknowns.

### Benefit to Clinician and Manufacturer: Detecting Abnormal Device Behavior

We next demonstrate a finding from the dataset which could be interpreted as a marker of either a failed fitting or abnormal behavior of the device. We modeled the data dimension “mean Input dB SPL” as a function of the fraction of time the device was in its directional mode. We expected that, as the mean Input dB SPL increased, representing a noisier environment, the device would spend more time using a directional rather than an omnidirectional mode. We were not privy to the exact internal workings of the device, but would expect that the decision to use the directional mode may also be influenced by the acoustic environment classifier, not just the Input SPL, as reported in some hearing-aid designs (Banerjee, 2011). Hence, our simplified assumption in this analysis, that there was a solid link between the directionality and the Input SPL, may not hold in all circumstances. The in-ear styles of hearing aid did not have directional microphones, so the focus was on a style that did, specifically a single model, the BTEa. With the device constraint, and after cleaning, the filtered dataset contained 62,277 entries.

The relationship between Input dB SPL and the time in directional mode was modeled as a regression using a Gaussian process. The Gaussian process framework is a flexible probabilistic approach. The principled probabilistic underpinnings of the framework provide a key strength of Gaussian processes; their ability to quantify uncertainty. By knowing the mean and variance of the process describing the data, we can set boundaries outside of which the existence of a data point can be regarded as rare, possibly indicating abnormal behavior. Due to the size of the filtered data, we use a sparse Gaussian process model with 100 inducing points (Mellor et al., 2018) and a kernel based on a combination of linear, squared-exponential, and bias (offset) components. The size of Gaussian process models normally grow linearly with the size of the training data. However, the full model can be approximated using inducing points which makes the model size proportional to the number of inducing points instead (Mellor et al., 2018). We use the GPy Gaussian process library (The GPy, 2012–2015).

**Figure 4.**
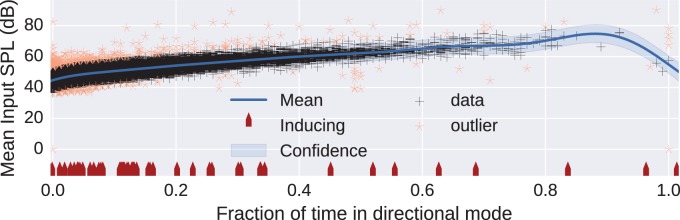
Gaussian process regression of the mean Input dB SPL encountered by BTEa devices as a function of the fraction of time spent in directional mode. The mean regression function and confidence interval between quantiles 0.1% and 99.9% are shown. The light amber stars outside of the confidence intervals are outliers which are considered to show abnormal behavior. The upward pointing arrows (red online) indicate the inducing points (Mellor et al., 2018) used in the analysis.

Figure 4 shows the scatter of data points for this analysis, with the mean shown by a solid line, and the (0.1% to 99.9%) confidence range shown as a shaded area about the mean. The position of the inducing points is shown on the abscissa as upward pointing arrows. The outliers in the dataset are shown by light stars (amber online). Some of these stars are distinctly separate from the main dataset and might warrant further investigation: Why would a hearing device spend 50% of its time in directional mode when the mean input level is very low (ca 40 dB SPL) or why would an aid being operated in a high sound level not spend much time in its directional mode? Some of the anomalous fittings may have occurred because pediatric fittings tend to have directionality disabled. Exclusion of all fittings with “Fracton of time in directional mode” equal to zero and rerunning the analysis did not make any major change to the final regression.

**Figure 5.**
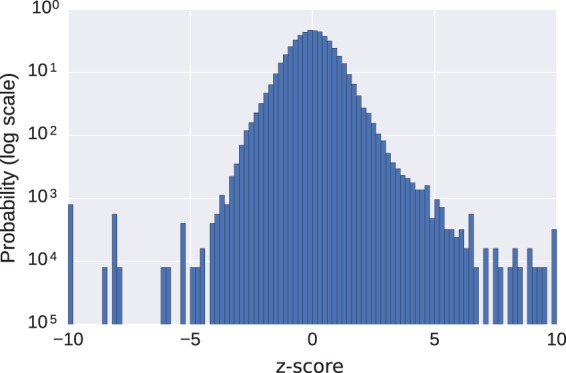
A histogram of the *z*-score ((value − mean)/standard deviation) of the data points shown in Figure 4, the mean Input SPL encountered by BTEa devices as a function of the fraction of time spent in directional mode. The mean and standard deviation for each example was determined by the Gaussian process regression model. The end bins include all examples exceeding ± 10 standard deviations from the mean.

Figure 5 shows the distribution of *z*-score of the mean Input SPL for each example in the dataset. The *z*-score of an example with value *v* is given by $\frac{v-\mu }{\sigma }$ where *μ* is the mean value and *σ* is the standard deviation of the value. The mean and standard deviation of the Gaussian process regression model evaluated at the fraction of time in directional mode was used as the mean and standard deviation in computing the *z*-score. The calculated *z*-score of the mean Input SPL for an example is with respect to the fraction of time the example was in directional mode. The end bins of the histogram contain all examples for which the *z*-score exceeded ±10. We can see that the distribution appears to be skewed, possibly partly due to the lowest category of Input SPL being <40. Although Gaussian models implicitly assume a lack of skewness, skewness at higher *z*-scores is presumed not to greatly influence the reliability of the modeling. Since *z*-scores exceeding ±5 should occur less than once in a million examples, the high rate of occurrence in a sample size of 62,277 implies that there is a population of fittings that merit further investigation in order to understand the cause. Although there may be valid reasons for these outliers, detecting such possibly abnormal behavior, and the reasons behind them, might be relevant to allow manufacturers to detect defective operation either due to device failure or unexpected programming by the clinician. In this particular example, only around 0.2% of all fittings fall into this category of “abnormal,” so may not represent a major concern to a clinical workload but is included as a didactic example of data mining.

### Benefit to the Patient: Three Extensions of Gatehouse et al. (2006a, 2006b)

The studies of Gatehouse et al. (2006a, 2006b) established links between factors other than degree of hearing loss that characterized the patient, but had not traditionally been considered part of the fitting process of a hearing aid. The purpose of the following analyses was to look for similar links between the patient and their use of their device. As outlined in Mellor et al. (2018), there could be many or few significant clusters of relevance to each analysis. Since we were trying to establish proof-of-concept for the techniques of data mining, for modalities with an ordinal scale, we chose to search for a fixed number (five) of clusters to give a sample picture of the data. For modalities with a cardinal scale, such as device style, each category became a cluster center (six for device style).

#### Linking device style to use

In the study of Gatehouse et al. (2006b), the social lifestyle of the patient was explored by the wearing of a comparatively bulky sound level dosimeter (Quest Technologies Q-400). This dosimeter could record sound levels at short intervals over long timescales, but its battery life and memory was limited, requiring frequent servicing for battery replacement or data transfer. The dimension “InputSPL” logged in the hearing aid, and present in the dataset, performs a similar function to the dosimeter, if not at the fine-grained timescale of those used by Gatehouse et al. (2006b), and also in a coarser categorization of input level into bins with 5-dB spacings. We interpret that the dimension “InputSPL” offers a glimpse into the acoustic demands on the user. Gatehouse et al. (2006b) used the phrase “auditory lifestyle or ecology” to describe their summary measures because they were linking both sound level and, via a diary and questionnaires, the perceptual demand in those environments. A perceptual demand measure was not available to us. We therefore interpret that, if the device is commonly used toward the middle and upper end of the range of Input SPL, the user has an acoustically demanding lifestyle spanning either work or leisure, or both. By this, we understand that a longer duration logged with higher sound levels indicates that the user has to either be environmentally aware or need to communicate, rather than deactivate the aid. To avoid confusion of terminology with that of Gatehouse, we therefore use the phrase “acoustic demand.”

The first analysis considers the possible relationship between the device style and range of Input SPL to answer the question “Is a particular device style associated with a particular pattern of acoustic demand?” After cleaning (removing entries with missing values for device style or any of the Input SPL dimensions), only the most recent entry for each device was kept for analysis so that the dataset contained 250,258 entries. There were 12 dimensions for Input SPL leading to $2^{12}-1$ (=4095) possible combinations of dimensions in this modality. All combinations were searched. This leads to $(2^{12}-1)\times 5\times 6=122,850$ tests to adjust for using the Bonferroni correction. This correction is noted in explanation of the algorithm in the subgroup discovery section of a companion paper (Mellor et al., 2018). The resulting threshold (desired *p*-value/number of tests) is therefore small. Such a search size does greatly increase the risk of false positives: Hence, we qualify the significance by setting a criterion on reporting results where the effect size was considered sufficiently “large” (1.1). Since some data dimensions, such as device style, were categorical (ITE, CIC, etc.), the clusters were centered on the styles themselves. There were eight dimensions for the audiogram.

**Figure 6.**
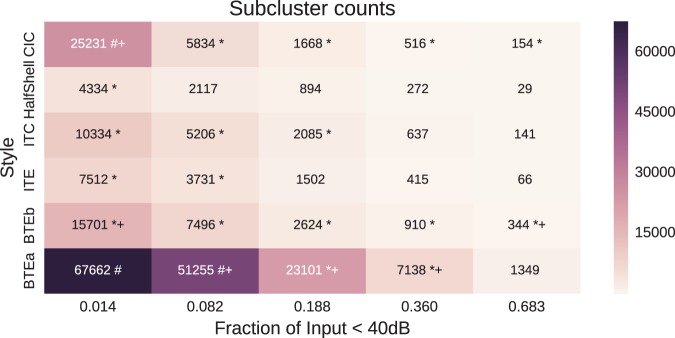
Interesting subgroups between device style and proportion of time the Input SPL was below 40 dB. The righthand side of the figure presents the heatmap according to number of devices. The lefthand side shows the number of devices in each subgroup. The abscissa labels represent the centers of the five clusters that were being sought. The interesting subgroups that are less than 0.1 of the population are marked with a * and those over 0.1 are marked with a #. All subgroups with an effect size over 1.1 are marked with a +.

The subgroup discovery method of Umek and Zupan (2011) was used and an example of the subgroup found which contained the largest effect size is shown in Figure 6.

This subgroup occurred when the Input SPL modality contained only a single dimension, the proportion of time spent below 40 dB SPL (a very quiet environment or “the bottom drawer”).

Here, the value of the Input SPL <40 dB cluster refers to the cluster center, so the subgroup (BTEa, 0.188) refers to the subgroup containing the device style BTEa and Input SPL <40 dB cluster with center 0.188.

There are two interesting subgroups that have an effect size exceeding 1.1 and are greater than 0.1 of the entries in the dataset. These correspond to the subgroup (0.014, CIC) and (0.082, BTEa). Other interesting subgroups where the effect size exceeded 1.1, but constituted a smaller share of the population, were (0.188, BTEa) and (0.360, BTEa).

Despite there being many examples of BTEa devices in the dataset, their use is commonly associated with two clusters: more common for a low amount of Input below 40 dB SPL (0.082), and less common for moderate occupancy of low-sound-level environments (0.188 and 0.360). However, the use of the CIC is more common where it is rare (0.014) to operate with an Input SPL of less than 40 dB. We associate this latter group with a higher acoustic demand (or the aid spends less time in a drawer than for other device styles). The phenotype of acoustic demand therefore appears to have a strong link with device type.

To test the validity of such a finding, we next expanded the dimensionality of the Input SPL data from the use of a single value to the use of the full 12 values, in an attempt to predict the choice of device style from a more complex classification of the patient’s acoustic demand. To this end we ran a 10-fold cross-validation of a Random Forest containing 50 trees to predict device style from the Input SPL range modality. The trees were split according to the Gini impurity (Mellor et al., 2018) where branch depth was set as the default of the Pedregosa et al. (2011) package used.

**Figure 7.**
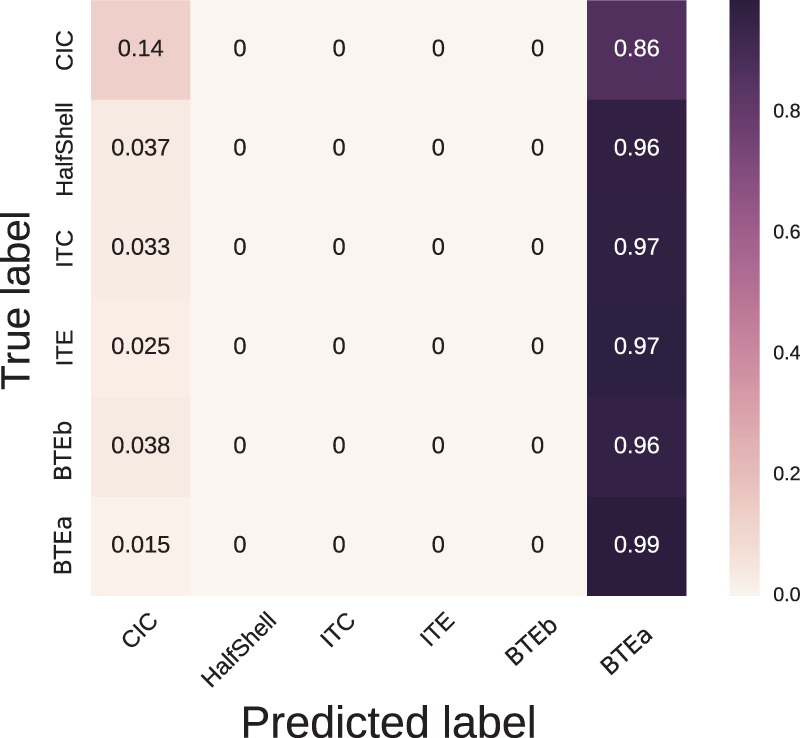
The confusion matrix generated from 10-fold cross-validation of a Random Forest classifier predicting device style from the Input SPL modality.

The confusion matrix is shown in Figure 7. This compares the device that would be predicted to be used (abscissa category), on the basis of the Input SPL modality, against the device actually used (the ordinate category). The cumulative prediction from the “leaves” of the individual trees in the Random Forest are therefore summarized in each cell of the matrix. A summary of this matrix is that, nearly independent of the device actually used, the Random Forest model would predict use of a BTEa device (dark column). This would imply that, regardless of acoustic demand phenotype, it is likely that a BTEa was used. At first sight, this indicates that the predictive power of the Input SPL modality was poor, contradicting our earlier finding. The reason for this poor predictive power is due to the ubiquity of the BTEa device dominating the dataset. The prediction of the learned Random Forest showed much better performance on training data, which suggested overfitting. We therefore trained another Random Forest model with the same settings except for setting the maximum branch depth before reaching a leaf to 4. This would prevent the Random Forest from learning rules too specific to individual examples. In this case, the decisions learned always predicted BTEa regardless of input. This was true for both the testing and training data in the cross-validation. Despite the tendency to predict BTEa regardless of input, we can glean useful information from the Random Forest.

**Figure 8.**
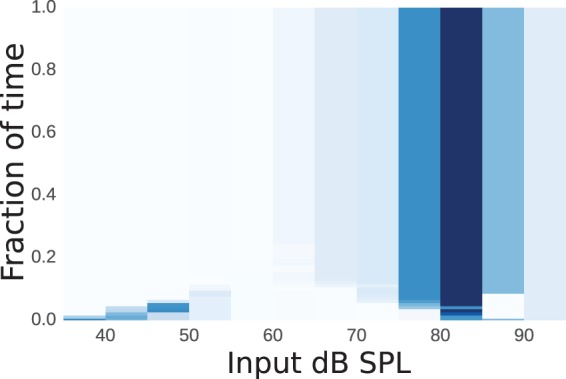
A heatmap showing the areas of the Input SPL modality that predicted a CIC device. Darker hues (of blue online) denote areas of high predictive power and white denotes areas of low predictive power.

We can see from the confusion matrix that prediction of use of the CIC device was most easily discriminated from that of the BTEa device by the Random Forest method (top left-hand corner of the matrix, cell value of 0.14). If all of the leaves correspond to a similar subspace of the Input SPL dimension, this hints toward a dependence between this subspace and the CIC device. Figure 8 shows a heatmap of the subspaces corresponding to a prediction of use of a CIC over all decision trees learned in the cross-validation of the Random Forest. White areas of the map show areas of low predictive power and darker areas show areas of high predictive power. We can see that CIC devices were predominantly predicted either when the device was used infrequently with Input SPL below 40 dB or also when the device was used more frequently in the range 80 dB to 85 dB. This would imply that CIC devices were used by people with higher acoustic demands. This corresponds to the earlier findings of the subgroup discovery. We therefore have shown two different approaches leading to the same finding. This correspondence in result gives us confidence that the finding is a reflection of the dataset and not an artifact of the method used.

From this, we suggest that CIC devices may be recognized as achieving greater market penetration than the use of BTEa devices for those people with a higher acoustic demand. Although anecdotes from experienced clinicians may cover such a suggested link, the information is carried with some robustness within a large dataset collected only over a few years, not a lifetime. Of course, other explanations are possible such as:

- a circular argument exists where the clinician recommends a CIC based on “perceived wisdom,” and
- given the increased price of a CIC over a BTE due to the customization required, there may be a contribution of other, psychological, factors of which price and cosmetic appearance are just a few.

Exploration of such would require a richer dataset.

#### Linking audiogram to device use

**Figure 9.**
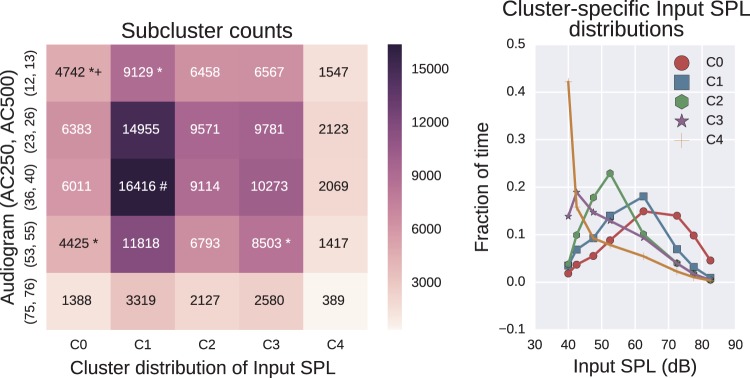
Interesting subgroups between audiogram thresholds at 250 and 500 Hz (AC250 and AC500) and proportion of time in given Input SPL ranges. The left plot shows the counts for each subgroup. The right plot shows the relative weights of the five Input SPL clusters. Each of the five plots shows the fraction of time the mean cluster member was in each selected Input SPL level. The interesting subgroups that contain less than 0.1 of the population are marked with a *. No interesting subgroup contained greater than 0.1 of the population. All subgroups with an effect size over 1.1 are marked with a +.

We next considered possible interactions between the audiogram data and Input SPL. After cleaning, the dataset contained 157,898 entries. There are 12 dimensions for the Input SPL leading to $2^{12}-1$ possible combinations of dimensions in this modality. There are eight dimensions for the audiogram and therefore $2^{8}-1$ possible combinations of dimensions for the audiogram modality. All combinations were searched. This leads to $(2^{8}-1)\times (2^{12}-1)\times 25=26,105,625$ tests to apply, allowing for the effects of multiple comparisons using the Bonferroni correction. Such a large search space does greatly increase the risk of false positives: Hence, the earlier qualification with a criterion on effect size. Additional sanity checks for false positives are typically included in the “Interpretation/Evaluation” section of the KDD process. The largest effect size occurred when the Input SPL modality contained all 11 dimensions and the audiogram modality contained just 2 of the 8 possible dimensions, the thresholds at 250 Hz (AC250) and 500 Hz (AC500). The results are shown in Figure 9.

The audiogram clusters are denoted by the corresponding cluster centers (in dB HL) and the traces of the higher dimensional Input SPL clusters are labeled and shown on the right-hand plot. These show a general pattern from clusters C0 to C4 of patients who spend progressively less time in environments with moderate to high sound levels. No interesting subgroup had a size exceeding 0.1 of the total population (after cleaning). An interesting subgroup, where the effect size exceeded 1.1, was (12,13, C0) as given by Figure 9. This corresponds to users with good hearing in lower frequency ranges (250 to 500 Hz) who habitually occupy louder environments. This pattern hints at a progressively lower acoustic demand as the degree of hearing loss increases. As previously, we qualify this statement by acknowledging that there may be other contributing factors to the finding such as age (which contributes to the degree of hearing loss (International Organization for Standardization, 2000)) for which, in this dataset, we could not control.

#### Linking environmental gain reductions to use

**Figure 10.**
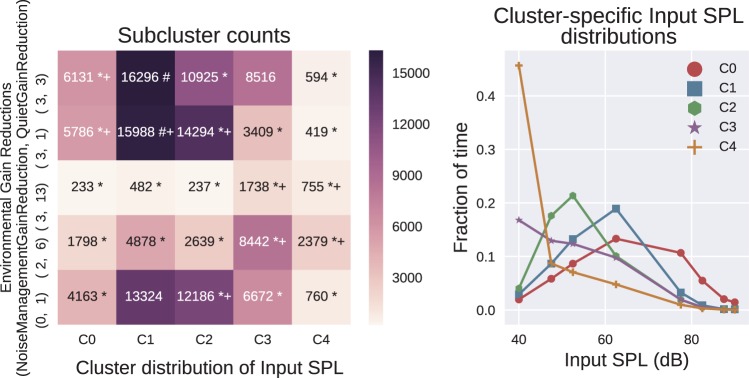
Interesting subgroups between noise-management + quiet gain reduction and the fraction of time spent in a subset of the Input SPL intervals. The left plot shows the counts for each subgroup. The right plot shows the five Input SPL clusters. Each of the five plots shows the fraction of time the mean cluster member was in each selected Input SPL level. The interesting subgroups that are less than 0.1 of the population are marked with a * and those over 0.1 are marked with a #. All subgroups with an effect size over 1.1 are marked with a +.

Finally, we considered the interaction of Input SPL with a pair of modalities labeled as “environmental gain reductions,” reductions available either when the aid is classifying that it is operating in noisy or in quiet acoustic environments. After cleaning, the dataset contained 142,023 entries. This leads to $(2^{2}-1)\times (2^{12}-1)\times 25=307,125$ tests with the significance level being adjusted using the Bonferroni correction. Results are shown in Figure 10, in a similar style to those presented in Figure 9. Again, the right-hand panel shows a similar pattern of Clusters C0 to C4 indicating groups of patients who, as the cluster number increases, spend progressively less time in environments with moderate to high sound levels.

In the left-hand panel, in general, we can see that subgroups containing Input SPL clusters for the mostly quiet environments (C3 and C4) correspond to environmental gain reduction clusters with the largest quiet gain reduction. Interesting subgroups containing Input SPL clusters in moderately louder environments correspond to environmental gain reduction clusters with smaller quiet gain reduction.

## Discussion

Using a dataset that had been heavily redacted in order to maintain anonymity of the clients and the clinicians, we have shown the existence of significant relationships across modalities in data logged in hearing instruments. Traits of the device (the style) and the user (the audiogram) were linked to their acoustic demand (measured as the Input SPL of the device). These data modalities stretch across different domains.

The redaction, as well as our arms-length relationship with the anonymous manufacturer, has meant that the findings may be explicable by factors to which we were not privy and may be much more prosaic. Because of the extensive searching, some of the findings may exist purely by chance. Such uncertainties may only be resolvable by access to commercially sensitive information, and therefore have to be performed “in-house.” The work reported here should be considered as a “proof-of-concept” of the potential for data mining of such large datasets. Although we initially wished to look at the evolution of data between successive visits, the current level of access and obfuscation did not reliably permit such use. We are given to understand that the full dataset contained much more detail, so we speculate that this may help clarify such a use.

The automated analysis has identified patterns in the data that may warrant further investigation of:

- *dispensing behavior, for example, where the audiogram recorded in a custom-fit style exhibits marked changes between visits*: Although there may be a valid clinical explanation for a fluctuating hearing loss, such as Ménière’s disease, it may be indicative of other problems such as off-protocol programming or device failure, contributing to a potentially dissatisfied customer.
- *device performance*: The quality control of a device depends on spotting out-of-specification product (by its distinctly “non-average” use or operation). This can be due to many reasons, including dispensing behavior.
- *user performance and preference*: Is the device enhancing, maintaining, or degrading the previous (or preferred) lifestyle of the user?

The collection of data modalities much wider than just the hearing loss of the patient was the philosophy behind Gatehouse et al. (2006a, 2006b), but the results presented here have extended beyond the patient (iii above) to include the dispensing clinician (i, above) as well as the manufacturer (ii, above), that is, including more of the stakeholders in the hearing-aid fitting process. One issue is the establishment of a baseline behavior of the patient before the intervention has started. This is where the predictive power of the data mining is required. Can demographic factors act as a proxy for the anticipated behavior, right from the time of first fitting?

On richer datasets, the techniques appear to offer great promise to personalize fittings from an earlier stage in the patient journey, as well as the possibility to automatically fine-tune the device based on usage patterns, which can be done without return to the clinic.

Such fine-tuning would benefit from a more reliable sense of individual success. The dimensions we analyzed here would need to be augmented with richer information such as the user’s report of benefit or satisfaction. To avoid clinician overload, such data should be collected from other sources routinely generated in the client care pathway (e.g., the clinical record of standard-procedure questionnaires, number of revisits, reason for visit, and attendance patterns).

Finally, after such conclusions, it serves to be reminded that the datasets are a collection of numbers, exhibiting statistical relationships (and flaws). These relationships only acquire importance with interpretation by, and the attachment of meaning from, professionals such as audiologists and device designers. Only then will the spurious be separated from the causal relationships. Perhaps there exists other categories between “causal” and “spurious,” which we tentatively label as “novel” or “unforeseen,” which prompt research questions to define more conventional, such as laboratory-based, experiments.
